# What can be learned from the residual efficacy of three formulations of insecticides (pirimiphos-methyl, clothianidin and deltamethrin mixture, and clothianidin alone) in large-scale in community trial in North Benin, West Africa?

**DOI:** 10.1186/s12936-023-04572-9

**Published:** 2023-05-08

**Authors:** Esdras Mahoutin Odjo, Albert Sourou Salako, Germain Gil Padonou, Boulais Yovogan, Constantin Jésukèdè Adoha, Bruno Adjottin, André Aimé Sominahouin, Arthur Sovi, Razaki Osse, Casimir D. Kpanou, Hermann W. Sagbohan, Armel Djenontin, Clement Agbangla, Martin C. Akogbeto

**Affiliations:** 1 Centre de Recherche Entomologique de Cotonou, Cotonou, Benin; 2 Faculté des Sciences et Techniques de l′Université d′Abomey-Calavi, Abomey-Calavi, Benin; 3 Department of Disease Control, Faculty of Infectious and Tropical Diseases, London School of Hygiene and Tropical Medicine, London, WC1E 7HT UK; 4 Faculté d′Agronomie de l′Université de Parakou, Parakou, Benin; 5 Université Nationale d’Agriculture de Porto-Novo, Porto-Novo, Benin; 6 Direction Générale de la Recherche Scientifique, Ministère de l’Enseignement Supérieur et de la Recherche Scientifique, Cotonou, Benin

**Keywords:** *Anopheles*, Indoor residual spraying, Wall surface type, Integrated vector management, Malaria

## Abstract

**Background:**

In Alibori and Donga, two departments of high malaria incidence of Northern Benin, pirimiphos-methyl, mixture deltamethrin + clothianidin, as well as clothianidin were used at large scale for IRS. The present study aimed to assess the residual efficacy of these products.

**Methods:**

Immatures of *Anopheles gambiae *sensu lato (*s.l*.) collected in the communes of Kandi and Gogounou (Department of Alibori), Djougou and Copargo (Department of Donga) were reared until adulthood. Females aged 2–5 days were used for susceptibility tube tests following the WHO protocol. The tests were conducted with deltamethrin (0.05%), bendiocarb (0.1%), pirimiphos-methyl (0.25%) and clothianidin (2% weight per volume). For cone tests performed on cement and mud walls, the *An. gambiae* Kisumu susceptible strain was used. After the quality control of the IRS performed 1-week post-campaign, the evaluation of the residual activity of the different tested insecticides/mixture of insecticides was conducted on a monthly basis.

**Results:**

Over the three study years, deltamethrin resistance was observed in all the communes. With bendiocarb, resistance or possible resistance was observed. In 2019 and 2020, full susceptibility to pirimiphos-methyl was observed, while possible resistance to the same product was detected in 2021 in Djougou, Gogounou and Kandi. With clothianidin, full susceptibility was observed 4–6 days post-exposure. The residual activity lasted 4–5 months for pirimiphos-methyl, and 8–10 months for clothianidin and the mixture deltamethrin + clothianidin. A slightly better efficacy of the different tested products was observed on cement walls compared to the mud walls.

**Conclusion:**

Overall, *An. gambiae s.l.* was fully susceptible to clothianidin, while resistance/possible resistance was observed the other tested insecticides. In addition, clothianidin-based insecticides showed a better residual activity compared to pirimiphos-methyl, showing thus their ability to provide an improved and prolonged control of pyrethroid resistant vectors.

## Background

Indoor residual spraying (IRS) and long-lasting insecticidal nets (LLINs) are the main strategies used to control and eliminate malaria [1]. They are generally effective when well implemented [2]. Scaling-up the implementation of these strategies has been associated with significant reductions in malaria-related morbidity and mortality between the years 2000 and 2015 [3]. Indeed, Millennium Development Goal number 6, which included reversing the incidence of malaria by 2015, has been achieved [4]. Unfortunately since 2015, progress has stalled [5]. One of the main reasons is the widespread and increasing resistance of malaria vectors to insecticides [6]. Indeed, vector resistance to insecticides can seriously jeopardize the effectiveness of vector control tools [7, 8]. Between 2010 and 2020, 89% of countries that provided data globally, reported resistance to at least one insecticide class in at least one malaria vector, while 33% detected resistance to pyrethroids, carbamates, organophosphates and organochlorines across different sites and 22% have confirmed resistance to all these 4 classes of insecticide in at least one site and at least one local vector [9]. In Benin, IRS started in 2008. From 2008 to 2016, three insecticides (bendiocarb 800 g/kg, pirimiphos-methyl 50 EC and pirimiphos-methyl 300 CS) were used and replaced due to their short residual efficacy on the walls. Indeed, due to the emerging pyrethroid resistance across Africa [10, 11], bendiocarb 800 g/kg (carbamate) was evaluated [12], then selected for a large-scale IRS from 2008 to 2012 in the Ouémé, and Atacora departments [13]. The emergence of resistance of *An. gambiae s.l*. to this product, as well as its low persistence (3 months) on the wall, after only 2 years of use in IRS have been reported in some areas of Benin region [14–17]. This result led to its replacement by pirimiphos-methyl 50 EC in 2013, followed by pirimiphos-methyl 300 CS (organophosphate) between 2014 and 2019. Cone tests performed with all these insecticide formulations showed a residual activity ≤ 5 months, while the transmission period occurred over 7 months. Thus, to comply with the WHO recommendations regarding resistance management [18], and have a new cost-effective product for IRS, the mixture clothianidin 500 g/kg + deltamethrin 62.5 g/kg (neonicotinoid + pyrethroid), and clothianidin 50 WG alone were used in 2020 and 2021 respectively, to replace pirimiphos-methyl 300 CS in the large-scale IRS campaign implemented in the two targeted departments. Clothianidin, a new insecticide belonging to the class of neonicotinoid, is one of the hopeful molecules at the moment. It was added to the WHO pre-qualification list for use in IRS [19] after showing good performance against resistant vector populations in laboratory, semi-natural environment [20, 21], and on a small scale in the community [22, 23]. Do new generation clothianidin-based insecticides have longer residual activity in the community and on a large scale than the traditional neurotoxic ones?

The present study was conducted to evaluate the residual efficacy of pirimiphos-methyl 300 CS, clothianidin + deltamethrin mixture (56.25 WP) and clothianidin 50 WG used in the large-scale IRS implemented in the Alibori and Donga departments from 2019 to 2021.

## Methods

### Study area

**Fig. 1:**
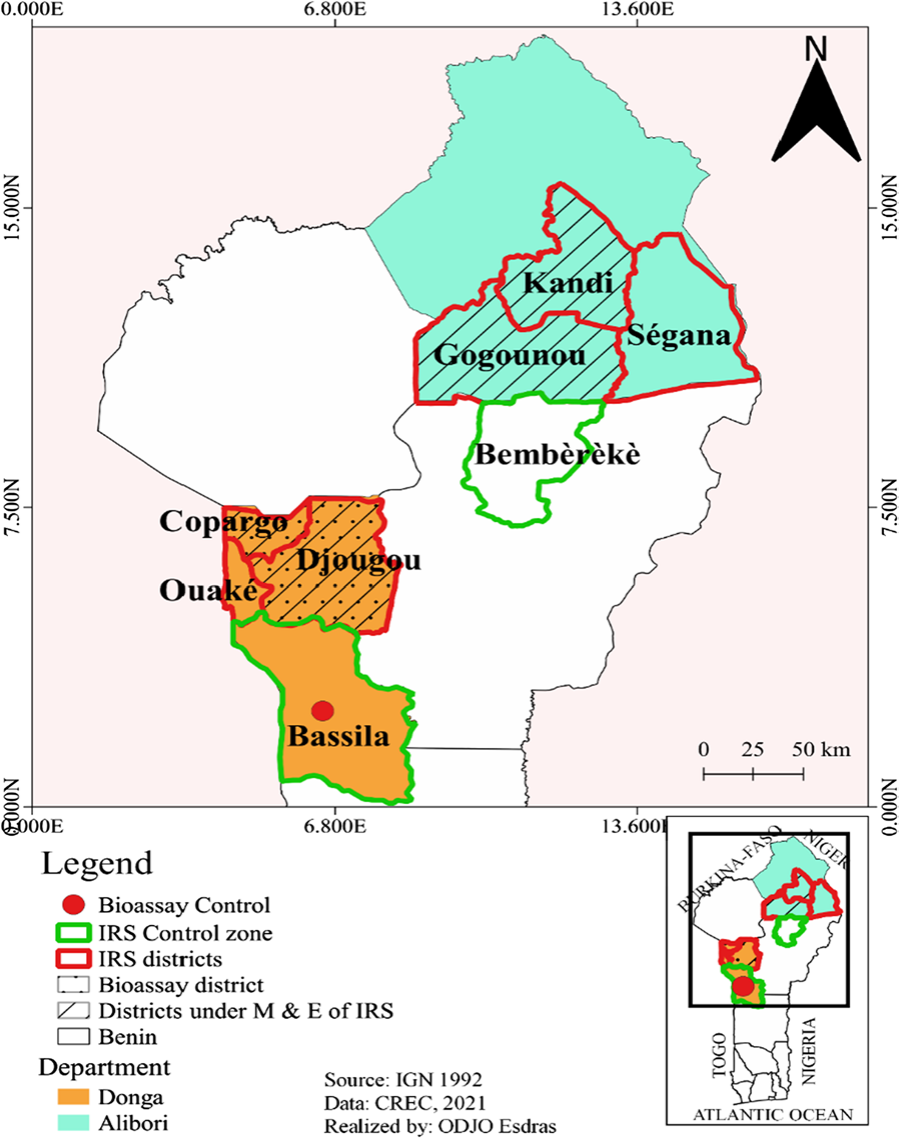
Map of the study area

The departments of Alibori and Donga are characterized by the Sudanian and Sudano-Guinean climate, respectively [24, 25]. Both departments have two seasons, one rainy and the other dry. In the Donga department, the dry season lasts from mid-October to mid-April, and the rainy one the rest of the year. Rainfall is between 1200 mm and 1300 mm, with August as the wettest month. The vegetation is dense along the watercourses forming gallery forests. Classified forests occupy more than half of the area of the commune of Bassila. In the commune of Djougou, wooded or shrubby savannahs are found and a small part occupied by classified forests [25]. In the Alibori department, the rainy season lasts 5–6 months (May to October) with rainfall ranging between 700 and 1200 mm. Sparse shrubby savannahs, highly degraded grassy tree savannah, gallery forests that run along the rivers, as well as valleys are also present [24] (Fig. 1).

### IRS campaigns

The IRS campaigns implemented in Alibori and Donga with pirimiphos-methyl 300 CS in 2019, and mixture clothianidin 500 g/kg + deltamethrin 62.5 g/kg in 2020, targeted 335,978 structures with the aim of protecting 1,112,610 people [26]. Overall, 335,207 structures representing a coverage rate of 86.5% were sprayed in the communes of Djougou, Copargo, Ouaké in the department of Donga, and Kandi, Gogounou, Segbana in the department of Alibori. This enabled the protection of 1,077,411 people (551,157 in Donga and 526,254 in Alibori) including 243,648 (22.6%) children under 5 years old (123,424 in Donga and 120,224 in Alibori) and 51,872 (4.8%) pregnant women (28,703 in Donga and 23,169 in Alibori) [26]. A total of 280,237 structures were sprayed in the two departments with clothianidin 50 WG, with 927,007 people protected in 2021. Compared to 2019 and 2020, a decrease in the coverage rate of 16.40% was observed in 2021, exposing more than 150,404 people to mosquito bites.

### Biological material

The local mosquito population of *Anopheles gambiae *sensu lato (*s.l*.) from the different IRS communes, and the susceptible strain of *An. gambiae* (Kisumu) were used for the WHO susceptibility tube and cone tests, respectively.

### Formulations of the evaluated insecticides

The three different insecticide formulations evaluated were pirimiphos-methyl 300 CS (organophosphate) in 2019, mixture 56.25 WP consisting of clothianidin 500 g/kg + deltamethrin 62.5 g/kg (neonicotinoid + pyrethroid) in 2020, and clothianidin 50 WG (neonicotinoid) in 2021.

### Pirimiphos-methyl 300 CS (Actellic 300 CS)

Pirimiphos-methyl capsule suspension is a WHO pre-qualified insecticide suitable for IRS [27]. Pirimiphos-methyl 300 CS is an organophosphate insecticide used at the recommended dose of 1.0 g of active ingredient (ai)/m^2^, for the control of malaria vectors. The action of Pirimiphos-methyl on acetylcholinesterase causes the accumulation of acetylcholine whose receptors are kept in an open position in the synaptic cleft, which prevents the transmission of nerve impulses and results in the death of the insect by paralysis.

### Clothianidin 500 g/kg + Deltamethrin 62.5 g/kg (56.25 WP) (Fludora^®^ Fusion)

The wettable powder (WP) formulation containing clothianidin (500 g/kg) and deltamethrin (62.5 g/kg) is packaged in 100 g sachets, which can be diluted for use in indoor residual spraying. The application rate recommended by the World Health Organization (WHO) is 0.4 g of product/m^2^, which corresponds to 200 mg/m^2^ of clothianidin and 25 mg of deltamethrin/m^2^ [28]. Clothianidin is a neonicotinoid insecticide that acts on the central nervous system of insects by binding to the nicotinic acetylcholine receptor, thus causing the paralysis and death of the insect. Deltamethrin is a pyrethroid, a class of insecticide that irreversibly blocks voltage-gated sodium channels, allowing the propagation of nerve impulses.

### Clothianidin 50 WG (SumiShield^®^ 50WG)

Clothianidin, a neonicotinoid insecticide, is a new synthetic insecticide. It was pre-qualified by the World Health Organization Pesticide Evaluation Scheme (WHOPES) [28] in 2017. This insecticide is a slow acting one. The recommended dose for its application is 300 mg ai/m^2^.

Use of insecticides with novel mode of action such as clothianidin can help provide effective and prolonged control of pyrethroid resistant malaria vectors. These insecticides can also help manage insecticide resistance by preventing strong reliance on the four traditional neurotoxic insecticide classes commonly used in public health. Also, their mixture with an insecticide that has a differing mode of action, can also slow down the emergence of resistance.

### Selection of houses and description of key activities

Two communes (Djougou and Copargo) in the Donga department were selected to assess the IRS quality and the residual effect of the insecticide used. Thus, an urban village, as well as a rural one, were randomly chosen in each of the two communes, which equates to a total of four villages. In each village, 20 treated houses of which 10 with cement wall, and 10 with mud wall, as well as 4 untreated houses (2 with cement wall, and 2 with mud wall) having served as control, were used to assess the IRS quality. Thus, a total of 96 houses were surveyed in the two communes to assess the quality of IRS and the persistence of the insecticide used. The quality control of the IRS was performed 1 week post-IRS implementation, using the *An. gambiae* Kisumu susceptible strain. The treated houses in which the 72 h post-exposure mortality rate was lower than 98%, were replaced by others that met this criterion to better appreciate the residual effect of the insecticide. The monitoring of the residual effect was performed on a monthly basis. Thus, mosquito specimens of the susceptible *An. gambiae* Kisumu strain, were exposed to the wall for 30 min. Thereafter, the exposed mosquitoes were gently removed and transferred into cleaned paper cups, where they were provided with a 10% sweetened juice. Over the 3 days observation period, the temperature as well as the relative humidity in the testing room were 27 ± 2 °C, and 80 ± 10%, respectively. Mortality rates were recorded 24, 48 and 72 h post-exposure.

### Cone testing

The cone testing were conducted in 2019, 2020 and 2021, following the WHO protocol [29]. Four cones were placed on the various faces of wall at different heights (0.5 m, 1 m, 1.5 m and 2 m) of each structure (cement and mud) to be tested. In each cone, ten females *An. gambiae* Kisumu, aged 2–5 days were introduced and exposed to the walls for 30 min, then removed from the cones and transferred to sterile veiled cups on which cotton pads soaked in a 10% sweetened juice were placed to feed the mosquitoes. Among the communes chosen for the entomological monitoring of IRS, Djougou and Copargo were selected to assess the persistence of insecticides in the Donga Department. Mortality rates were recorded for each batch of mosquitoes and per type of structure after 24, 48 and 72 h of observation at 27 °C ± 2 °C and 80% ± 10% relative humidity. When control mortality was between 5 and 20%, the mortality was corrected using the Abottʼs formula. If the control mortality was > 20%, the test was repeated. If the mortality was < 5%, the test was considered valid and no correction was needed. The WHO efficacy threshold was 80%.

### WHO susceptibility tube testing

WHO susceptibility tube testing were performed in 2019, 2020 and 2021 to assess the susceptibility level of populations of *An. gambiae s.l.* collected from IRS communes. Thus, larvae were collected from natural breeding sites during the rainy season in Djougou, Copargo, Kandi and Gogounou. The collected mosquito larvae were transported to the CREC insectary where they were reared until adulthood. Emerged adult mosquitoes were provided with a 10% sweetened solution and kept under the following conditions: Temperature: 27 ± 2 °C, Relative humidity: 72 ± 5%. Non blood-fed females *An. gambiae s.l.* aged 2 to 5 days were used for WHO susceptibility tube testing. Deltamethrin (0.05%), bendiocarb (0.1%) and pirimiphos-methyl (0.25%) commonly used for vector control in Benin were tested.

With respect to the susceptibility of *An. gambiae* complex populations to clothianidin, it was only assessed in 2021 using the protocol optimized by Sumitomo Chemical Company (SCC) [30] in the absence of WHO guidelines for this purpose. The published WHO guidelines for susceptibility testing procedures for each pre-qualified insecticide allow Universiti Sains, a WHO collaborating institution located in Malaysia, to prepare the insecticide-impregnated papers at different doses and make them available to research centres [31]. Until 2021, the WHO had not published guidelines for clothianidin susceptibility testing.

Whatman papers cut to 12 cm wide by 15 cm long were impregnated with 13.2 mg of clothianidin active ingredient, and used for testing within 24 h of impregnation [30].

Mortality rates were recorded 24 h after exposure for deltamethrin 0.05%, bendiocarb 0.1%, pirimiphos-methyl 0.25%. However, the mortality rate for clothianidin was recorded on a daily basis over 6 days [30].

The susceptibility status of the tested mosquito populations, was determined according to the WHO criteria [32]:

- Mortality rate between 98 and 100%: susceptible mosquito population.
- Mortality rate between 90 and 97%: mosquito population with a possible resistance.
- Mortality rate below 90%: resistant mosquito population.

### Measured parameters

Four parameters were studied: susceptibility level of local populations of *An. gambiae s.l.* to insecticides, knock down effect or immediate mortality 30-min, delayed mortality, and residual efficacy [29].

### Statistical analysis

Data were processed simultaneously by Excel, Graphpad Prism 5 and R version 4.1.3. Graphs were made in GraphPad Prism 5 and Excel. The Chi-square test for comparison of proportions was used to determine the percentages of significance as well as the confidence intervals.

## Results

### Insecticide resistance status of *An. gambiae s.l.*

**Fig. 2:**
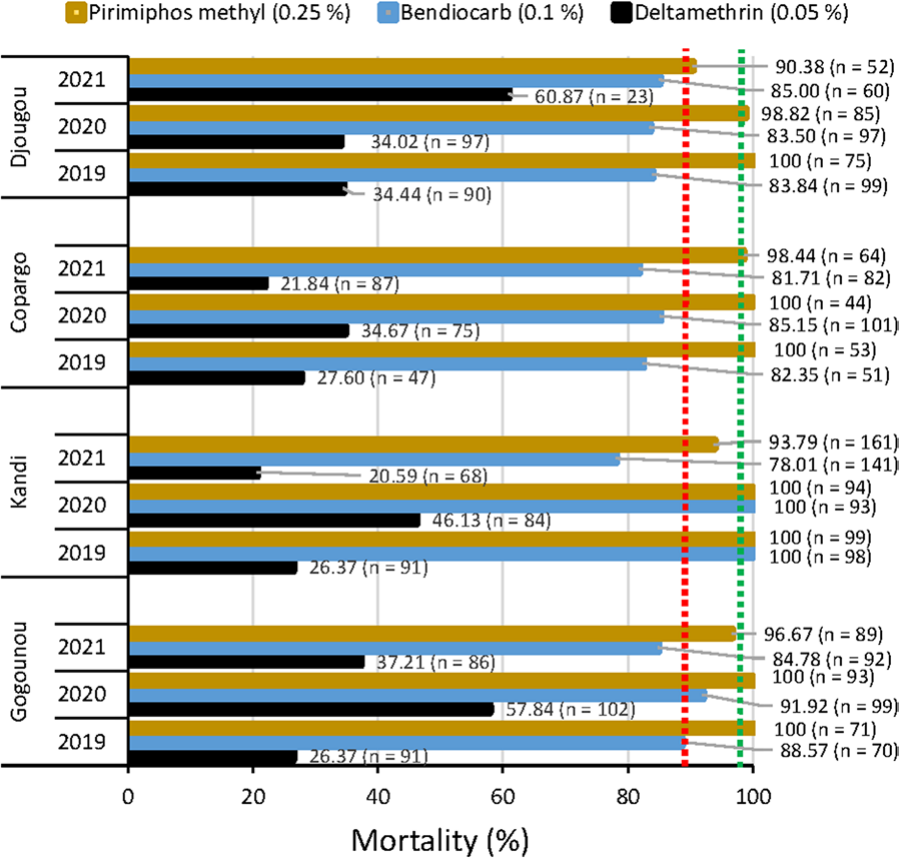
24 h mortality rates after exposure to deltamethrin 0.05%, bendiocarb 0.1% and pirimiphos-methyl 0.25%. The red dotted line indicate a 90% mortality rate, The green dotted line indicate a 98% mortality rate

The Fig. 2 shows the susceptibility level of *An. gambiae s.l.* to deltamethrin 0.05%, bendiocarb 0.1% and pirimiphos-methyl 0.25% over the three study years. Deltamethrin resistance (mortality < 90%) was observed within mosquito populations from all surveyed communes over the three monitoring years. The same trend was observed with bendiocarb 0.1%, though there was a susceptibility in Kandi in 2019 and 2020, and a possible resistance in Gogounou in 2020. Overall, all tested vector populations were susceptible to pirimiphos-methyl 0.25% (mortality > 98%) in 2019 and 2020. However, in 2021, local vector populations from Djougou, Kandi and Gogounou, displayed a possible resistance to this product (mortality < 90%) (Fig. 2).

**Fig. 3:**
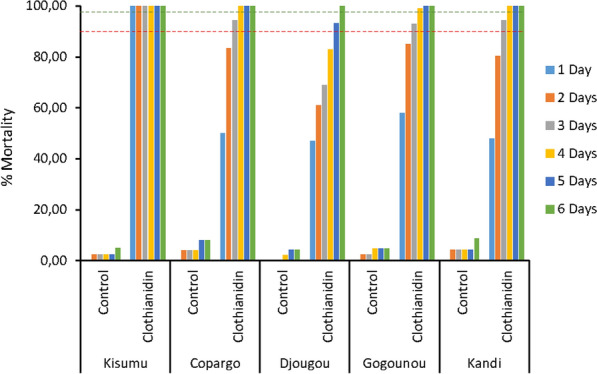
Mortality rates of *Anopheles gambiae* (Kisumu) and *Anopheles gambiae s.l.* F1 after exposure to clothianidin-treated Whatman papers. The red dotted line indicate a 90% mortality rate, The green dotted line indicate a 98% mortality rate

The mortality rate of the *An. gambiae* Kisumu reference strain after 24 h of exposure to clothianidin was 100% (Fig. 3). For field populations of *An. gambiae s.l*., mortality 24 h after exposure to clothianidin was 50% in Copargo, 47% in Djougou, 58% in Gogounou and 47.88% in Kandi. This mortality rate increased over time to reach mortality > 98% on the fourth day in Copargo (100%), Gogounou (99%) and Kandi (100%). It was not until 48 h later (day 6) that the mortality rate was above 98% in Djougou (100%) (Fig. 3).

### Efficacy of the three insecticide formulations on the susceptible *An. gambiae* Kisumu strain over the whole study area

**Fig. 4:**
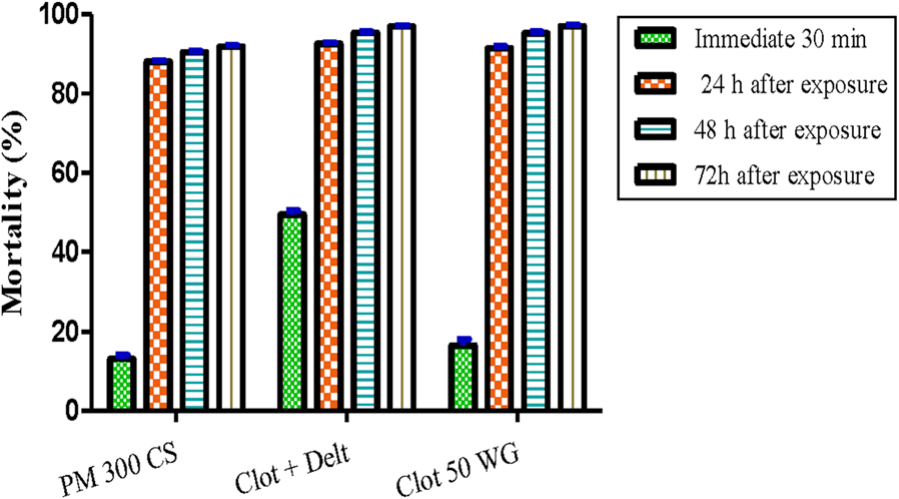
Mean mortality rates at different time points for the three tested insecticide formulations over the whole study period (combined data for both cement and mud walls). Pirimiphos-methyl 300 CS; (Clot + Delt) = mixture of Clothianidin 500 g/kg and Deltamethrin 62.5 g/kg; Clot 50 WG = Clothianidin 300 mg ai/m^2^; h = hour; % = percentage

More than 71,800 females *An. gambiae* Kisumu aged 2–5 days were exposed to the two types of treated walls (cement and mud). Mortality at different time points, of each insecticide formulation on the different types of wall were evaluated. Clothianidin-based insecticides showed a significantly better performance (*p* < 0.05) over the different time points compared to pirimiphos-methyl 300 CS. The mixture clothianidin 500 g/kg + deltamethrin 62.5 g/kg showed an immediate mortality rate that was 3 times higher than that of clothianidin 50 WG after exposure to the treated walls. No difference in the mean mortality rate was observed 48 h and 72 h post-exposure between the two clothianidin-based formulations (Fig. 4).

### Residual efficacy of insecticide formulations

**Fig. 5:**
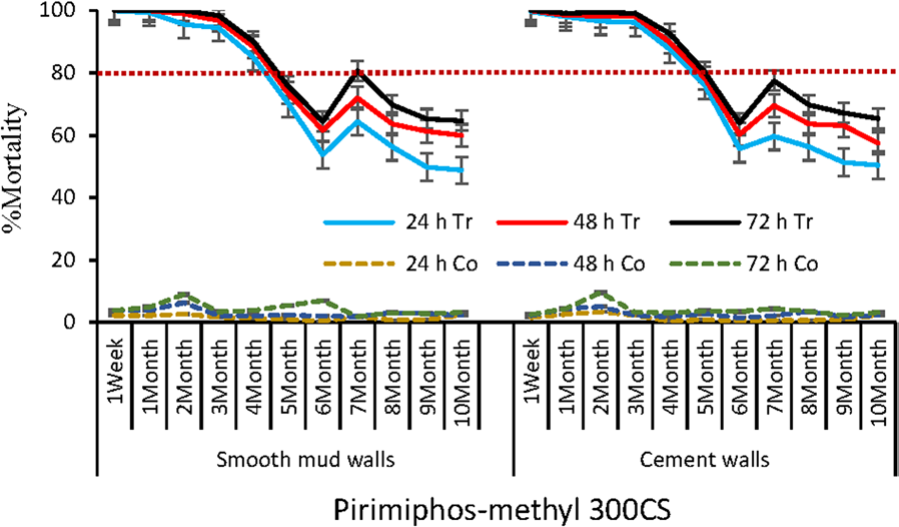
Residual efficacy of pirimiphos-methyl 300CS on mud and cement walls; h = hour; Tr = Treated rooms; Co=control rooms not treated with insecticides; % = percentage; The red dotted line indicate the WHO efficacy threshold of 80%

**Fig. 6:**
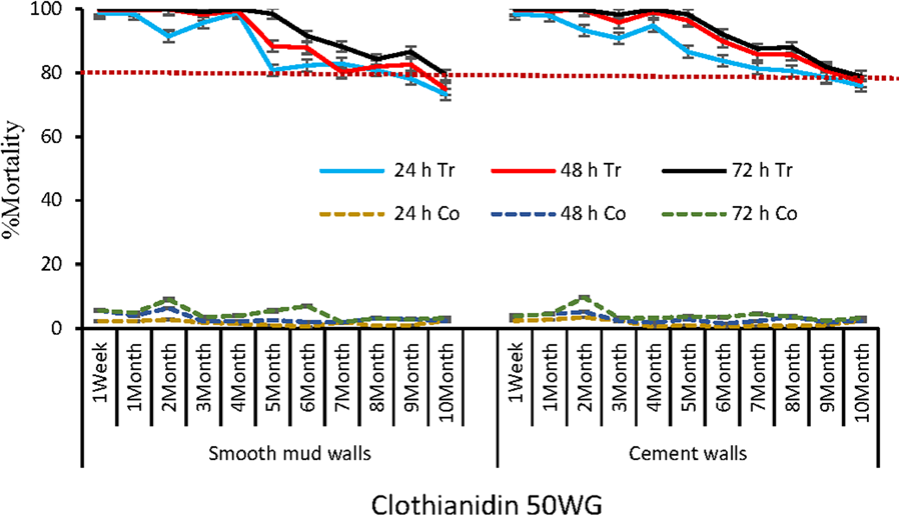
Residual efficacy of clothianidin 50WG on mud and cement walls. h = hour; Tr = Treated rooms; Co = control rooms not treated with insecticides; % = percentage; The red dotted line indicate the WHO efficacy threshold of 80%

**Fig. 7:**
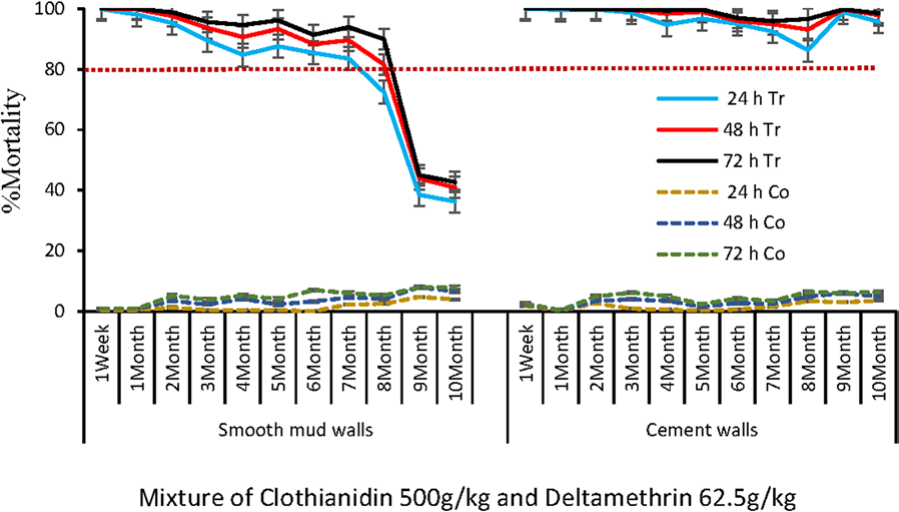
Residual efficacy of the mixture clothianidine 500 g/kg + deltamethrin 62.5 g/kg on mud and cement walls. h = hour; Tr = Treated rooms; Co = control rooms not treated with insecticides; % = percentage; The red dotted line indicate the WHO efficacy threshold of 80%

Results of the monitoring of the efficacy of the tested insecticide formulations are summarized in Figs. 5, 6, and 7. The three tested insecticide formulations are relatively more effective on cement walls than on mud ones. With pirimiphos-methyl 300 CS, an efficacy of 4 and 5 months was observed for mud and cement walls, respectively (Fig. 5). For clothianidin 50 WG, an efficacy of 9 and 10 months was observed for mud and cement walls, respectively (Fig. 6). With clothianidin 500 g/kg + deltamethrin 62.5 g/kg mixture, the efficacy lasted 8 months for mud walls, and 10 months for cement ones (Fig. 7). Although the mortality rates have increased over time points (24, 48, and 72 h post-exposure), this increase was not significant (Figs. 5, 6, 7).

## Discussion

In the present study, a large-scale community evaluation of the efficacy of three insecticide formulations used in IRS, was performed. The tested products were an organophosphate (pirimiphos-methyl 300 CS), a neonicotinoid (clothianidin 50 WG) and a mixture of neonicotinoid and pyrethroid (clothianidin 500 g/kg + deltamethrin 62.5 g/kg). Clothianidin-based insecticides are considered as potential alternative molecules to carbamates and organophosphates, products to which an emerging resistance is more and more observed.

WHO susceptibility tube testing revealed resistance of populations of *An. gambiae s.l.* from the four IRS districts to the diagnostic doses of deltamethrin. The same trend was previously observed by Salako et al. [33] and Kpanou et al. [34] in the same districts. This widespread resistance might be due to the distribution and massive use of LLINs, the uncontrolled use of pyrethroid insecticides in agriculture, and the domestic use of synthetic insecticides generally consisting of pyrethroids. The total loss of susceptibility of local vectors to bendiocarb (0.1%) in 2021, and the possible resistance to the diagnostic dose of pirimiphos-methyl were also reported by other trials in northern Benin [33, 35, 36]. This results in the replacement of bendiocarb 800 g/kg and pirimiphos-methyl 300 CS by clothianidin-based insecticides in Benin. Indeed, this replacement by an insecticide that has a new mode of action will help increasing the impact of IRS on local malaria vectors.

The mortality of more than 98% observed 24 h after exposure of the susceptible population of *An. gambiae* (Kisumu) to clothianidin (734 mg/m^2^) is the evidence of the lethal effect of this insecticide on mosquito vectors. The WHO susceptibility tube testing performed by exposing populations of *An. gambiae s.l.* from the study districts to the same product showed a delayed lethal effect, which was not observed with deltamethrin, bendiocarb and pirimiphos-methyl tested. Instead of an observation period of 24 h after exposure to the insecticide (standard WHO tube test), it took up to 4–6 days to observe a mortality of more than 98%. The same trend was previously observed by Oxborough et al. [30]. It might be appropriate to revisit the diagnostic dose as the longer the observation time, the more other factors could affect the mortality rate. Furthermore, an alternative WHO bottle bioassay recently described by Corbel et al. [37] could also be considered for evaluating the susceptibility of *Anopheles* vectors to clothianidin.

Cone testing performed throughout the study period, revealed higher mortality rates induced by clothianidin 50 WG and the mixture clothianidin 500 g/kg + deltamethrin 62.5 g/kg compared to that of pirimiphos-methyl 300 CS. Similarly, the immediate mortality recorded for the mixture clothianidin 500 g/kg + deltamethrin 62.5 g/kg was higher than that of clothianidin 50 WG, and pirimiphos-methyl 300 CS. The delayed mortalities induced by the three insecticide formulations on *Anopheles* mosquitoes increased over timepoints (24, 48, and 72 h post-exposure) but, without any significant difference (p > 0.05). However, the 24 h mortality induced by the mixture clothianidin 500 g/kg + deltamethrin 62.5 g/kg differed significantly from that of clothianidin 50 WG (χ^2^ = 12.744, df = 1, p = 0.0004). Between the two clothianidin based insecticides, the mixture clothianidin 500 g/kg + deltamethrin 62.5 g/kg performed better against susceptible *An. gambiae* Kisumu mosquitoes at 30 min and 24 h post-exposure, which could be due to the deltamethrin-induced immediate mortality which compensates the slow effect of clothianidin.

Both clothianidin-based insecticide formulations showed a residual effect of 10 months. This result confirms the performance previously demonstrated in the laboratory and in a semi-natural conditions [20, 38], then in a small-scale community [22, 23] against resistant vector populations. However, the residual efficacy of clothianidin observed in this study is lower (7 months) than that observed against *Anopheles culicifacies s.l.* in Gujarat, India [39]. The residual efficacy of clothianidin observed in this study is superior to that of the insecticides recommended for IRS by the WHO, so far [29]. The three insecticides induced performances (persistence, lethal effect) which varied according to the type of wall. This variation can be explained by the difference in porosity between the treated walls [40]. Indeed, the different types of walls do not absorb the insecticide solution in the same way and the bioavailability of the product on the substrates would therefore not be the same over time [41]. The relatively lower efficacy of all the insecticide formulations on the mud walls compared to the cement ones could be justified by the high porosity of the mud that are soil made. Indeed, several studies performed in experimental huts and in the community in Benin have shown that residual activity was often very low on highly porous substrates [42, 43].

## Conclusion

Overall, clothianidin-based insecticides have proven good efficacy and a better residual efficacy in public health compared to the first insecticides pre-qualified by WHO for IRS. The mixture clothianidin 500 g/kg + deltamethrin 62.5 g/kg, and clothianidin 50 WG showed superior efficacy over pirimiphos-methyl 300CS. Relatively higher mortality rates with the mixture clothianidin 500 g/kg + deltamethrin 62.5 g/kg compared to clothianidin 50 WG were observed 24 h post-exposure. The presence of deltamethrin in the mixture seems to have contributed to an improved efficacy of clothianidin. The results of an ongoing community trial assessing the impact of the two clothianidin based products on the entomological indicators of malaria transmission will enable a better assessment of their efficacy.

## Data Availability

The data used and/or analysed in this study are available from the corresponding author on reasonable request.

## Abbreviations

IRS: Indoor residual spraying
WHO: World Health Organization
LLINs: Long-lasting insecticidal nets
CREC: Centre de Recherche Entomologique de Cotonou
SCC: Sumitomo Chemical Company
CS: Capsule suspensions
WP: Wettable powders
WG: Water-dispersible granules
